# Genetic Variability of *Candida albicans* Sap8 Propeptide in Isolates from Different Types of Infection

**DOI:** 10.1155/2015/148343

**Published:** 2015-02-04

**Authors:** Joana Carvalho-Pereira, Catarina Vaz, Catarina Carneiro, Célia Pais, Paula Sampaio

**Affiliations:** Biology Department, Minho University, Campus de Gualtar, 4710-057 Braga, Portugal

## Abstract

The secreted aspartic proteases (Saps) are among the most studied virulence determinants in *Candida albicans*. These proteins are translated as pre-pro-enzymes consisting of a signal sequence followed by a propeptide and the mature enzyme. The propeptides of secreted proteinases are important for the correct processing, folding/secretion of the mature enzyme. In this study, the DNA sequences of *C. albicans* Saps were screened and a microsatellite was identified in *SAP8* propeptide region. The genetic variability of the repetitive region of Sap8 propeptide was determined in 108 *C. albicans* independent strains isolated from different types of infection: oral infection (OI), oral commensal (OC), vulvovaginal candidiasis (VVC), and bloodstream infections (BSI). Nine different propeptides for Sap8 processing were identified whose frequencies varied with the type of infection. OC strains presented the highest gene diversity while OI isolated the lowest. The contribution of the Saps to mucosal and systemic infections has been demonstrated and recently Sap8 has been implicated in the cleavage of a signalling glycoprotein that leads to Cek1-MAPK pathway activation. This work is the first to identify a variable microsatellite in the propeptide of a secreted aspartic protease and brings new insights into the variability of Sap8.

## 1. Introduction

*Candida albicans* adaptability has been attributed to several factors, including adhesion, phenotypic switching, hypha formation, and secretion of extracellular hydrolytic enzymes [1, 2]. Together, these factors contribute to the successful yeast colonization and promote resistance to immune system defences [3, 4].* Candida albicans* genome contains 10 secreted aspartic protease genes,* SAP1* through* SAP10* [5, 6].* SAP* genes encode pre-pro-enzymes consisting of a signal sequence followed by a propeptide and the mature proteinase domain. The prepeptide or signal peptide is necessary for entry into the secretory pathway by transporting the protein across the rough endoplasmic reticulum membrane [7]. This signal peptide is then removed in the endoplasmic reticulum and after folding the proenzyme is transported to the Golgi apparatus. Aspartic proteases are synthesized as inactive zymogens, inhibited by the presence of their N-terminal propeptides, which has been found to be essential for assisting the correct folding and secretion of its associated protein [8, 9]. Upon completion of folding, the propeptide is cleaved and removed to generate the active enzyme that in the case of* C. albicans* is through an exogenous proteolytic reaction in the Golgi apparatus dependent of the membrane-bound protease Kex2 [10–12].

The contribution of the Saps to mucosal and systemic infections and their involvement in adherence, tissue damage, and evasion of host immune responses has been demonstrated with* SAP*-deficient mutants and protease inhibitors [5]. Recent studies indicate little correlation between the expression of specific* SAP* genes and epithelial cell damage or infection, indicating that the proteinase family as a whole (Sap1–10) contribute to the infection [13, 14]. Saps have been shown to degrade a variety of host defense proteins such as lactoferrin and immunoglobulins [15] and E-cadherin, the major protein in epithelial cell junction [16].

Since its identification,* SAP8* expression in vitro has been detected at lower temperatures, 25°C, in culture medium [17], and in mucosal infection based on reconstituted human epithelium (RHE), although in late phases of the infection [18, 19].* SAP8* expression in vivo has been detected in murine, although transiently [20], and in human oral and vaginal infections although preferentially in vaginal rather than oral infections [5, 13]. However, its contribution to the infection process in humans appears to be minimal [13]. Recently,* C. albicans* Sap8 has been implicated in the proteolytic processing of Msb2 glycoprotein that allows Cek1 MAPK activation [21]. This MAPK pathway is involved in starvation-specific germ tube formation [22], responds to glycosylation defects in the cell wall [23], and modulates ß-glucan exposure on the cell surface, which in turn affects biofilm formation [24], and immune responses against* C. albicans* cells [25]. Sap8 has been identified as the most efficient aspartyl protease in Msb2 processing [21].

The mechanism by which Sap8 contributes to human mucosal infections is still unclear and requires more functional studies. Curiously, in this study we observed that* SAP8* contains a (CAA/G)_*n*_ microsatellite at the 5′end of the gene that codes for a poly-glutamine tract at the propeptide region of the protein. Sap8 was the only* C. albicans* secreted proteinase that presented a microsatellite, which was named CAVIII. Due to the key role of the propeptide in the folding and activity of the protease, the genetic variability of CAVIII microsatellite was to characterize in strains isolated from different types of infection.

## 2. Material and Methods

### 2.1. Yeast Strains

A total of 108* C. albicans* independent isolates were analysed in this study (Supplementary Table available online at http://dx.doi.org/10.1155/2015/148343). Twenty-six strains were isolated from saliva of patients diagnosed with oral infection, 30 from saliva of healthy patients, 28 from vulvovaginal infections, and 24 from blood cultures. Additionally, the type strains of* C. parapsilosis* (ATCC 22019),* C. krusei* (ATCC 6258),* C. tropicalis* (ATCC 750),* C. glabrata* (ATCC 2001),* C. bracarensis* (NCYC D3853),* C. guilliermondii* (ATCC 6260),* C. lusitaniae* (ATCC 34449),* C. dubliniensis* (CBS 7987),* C. orthopsilosis* (ATCC 96139), and* C. metapsilosis* (ATCC 96144) were also used.

### 2.2. Microsatellite Amplification and Allele Size Determination

A search in DNA sequences from all* Candida albicans* SAP genes, available in NCBI database, was performed in order to identify sequences containing microsatellite repeats. A sequence of (CAA/G)_10_ was identified in the SAP8 propeptide region of SC5314 strain and primers were designed for specific amplification. Amplification of this locus in all* C. albicans* strains analysed in this study was performed by colony-PCR as previously described [26] with Sap8 specific primers, CAVIII-F: 5′-TCCCTGAAGACATTGATAAAAGAGC-3′ and CAVIII-R: 5′-AGAATCAACCACCCATAAATCAGAA-3′. For automatic allele size determination, the CAVIII forward primer was 5′ fluorescently labelled with hexachlorofluorescein (HEX). PCR fragments were then separated in an ABI 310 Genetic Analyzer (Applied Biosystems Inc.) and fragment sizes determined automatically using the GeneScan 3.5 Analysis Software. The most frequent CAVIII alleles were sequenced using the procedure previously described [27]. All strains were also typed with CAI microsatellite [27]. CAI marker was selected because it is one of the most polymorphic loci for* C. albicans* strain differentiation and is located in a different chromosome, being independent from CAVIII. Only isolates with different multilocus genotypes were analysed in this study.

Specificity of CAVIII microsatellite was also assessed by testing DNA from other* Candida* clinically relevant species, such as* C. parapsilosis*,* C. krusei, C. tropicalis*,* C. glabrata*,* C. bracarensis*,* C. guilliermondii*,* C. lusitaniae*,* C. dubliniensis*,* C. orthopsilosis,* and* C. metapsilosis* with the primers designed and PCR conditions used in this study. Stability of CAVIII was also assessed comparing the results obtained after DNA extraction of two* C. albicans* strains grown over 300 generations, as previously described [27].

### 2.3. Clustering Analysis

Genetic distances between strains, based on the* SAP8* propeptide alleles, were calculated using the Shriver method (DSW distance) with the Populations1.2.30 software and clustering performed with NTSys2.0 software, by using UPGMA. Four groups of strains were defined, the VVC (28 strains from vulvovaginal candidiasis), the BI (bloodstream isolates, 24 strains), the OI (26 isolates from oral infections), and the OC (oral commensal, 30 strains).

### 2.4. Group Differentiation Tests

Allelic and genotypic frequencies were calculated and group differentiation tests were performed concerning allelic and genotypic distribution by testing the null hypothesis Ho: “the allelic/genotype distribution is identical across groups.” Considering microsatellite data, the significance of unbiased *P* values of the probability test for each group pair was estimated by using the Fisher method [28]. *P* > 0.05 indicates no significant differences were observed in the comparison between the two groups, and when *P* < 0.05 this indicates that there are significant differences. All these calculations were performed with Genepop4.1.3 software. Gene Diversity was calculated according to the following formula [29]:

(1) $$\begin{array}{c} \hat{H}=\frac{N}{N-1}\left(1-\overset{k}{\underset{i=1}{\sum }}p_{i}^{2}\right). \end{array}$$

## 3. Results

### 3.1. Microsatellite Analysis

**Figure 1:**
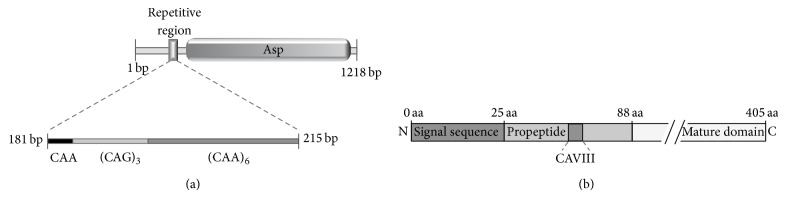
(a) Schematic representation of SC5314* C. albicans* SAP8 gene, showing the repetitive CAVIII region and the Asp (eukaryotic aspartyl protease) motif of the protein. (b) Representation of the Sap8 protein, showing the signal sequence (0 to 25 amino acids), the propeptide (26 to 88 aminoacids, with the microsatellite CAVIII within), and the mature domain (89 to 405 amino acids).

**Figure 2:**
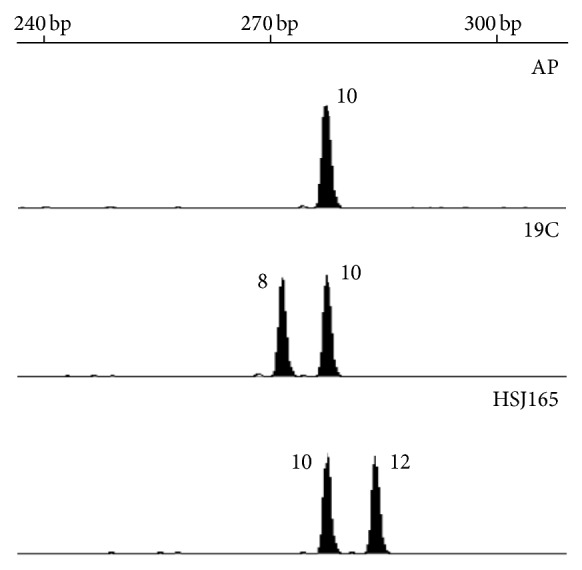
Representative GeneScan profiles. Electropherogram of three strains (AP, 19C, and HSJ165), showing the corresponding alleles. 240, 270, and 300 bp represent the section of the molecular weight scale where the CAVIII amplified fragments are located.

The analysis of the DNA sequences from all 10* C. albicans SAP* genes performed in this study identified a microsatellite region in the propeptide sequence of SAP8 gene (Figure 1(a)). The nucleotide sequence analysed from strains SC5314 (accession n^o^ XM_714848) presented a repetitive region (CAA/G)_10_ that codes for a tract of 10 glutamines within the Sap8 propeptide region (Figure 1(b)). Propeptides are considered to play a key role in the correct maturation of aspartic proteinases and thus the polymorphism of this microsatellite (named CAVIII microsatellite) was investigated in 108 independent clinical isolates. Nine different alleles and 14 distinct genotypes were identified.  Figure 2 shows an example of the allele and corresponding genotypes for three strains. This marker was revealed to be species specific, since no amplification products were obtained when CAVIII primers and PCR conditions described were used to amplify other pathogenic* Candida* species, namely,* C. parapsilosis*,* C. krusei*,* C. tropicalis*,* C. glabrata*,* C. bracarensis*,* C. guilliermondii*,* C. lusitaniae*,* C. dubliniensis*,* C. orthopsilosis,* and* C. metapsilosis*. Additionally, genomic stability of CAVIII microsatellite was confirmed by demonstrating the lack of size variations over 300 generations. Similar results have previously been reported for other* C. albicans* [30],* C. parapsilosis* [31], and* C. glabrata* [32] microsatellites. The reproducibility of CAVIII amplification was also confirmed by observing the same amplification fragments when comparing the results obtained with different colonies from the same strain obtained in different days. This analysis was performed for at least 5 different strains.

Sequencing of the most frequent fragments confirmed CAVIII locus specific amplification and allowed the determination of the number of repeated units for each fragment amplified (Table 1). The alleles obtained contained from 5 to 14 repetitive units, corresponding to the number of glutamines that will be present in the propeptide. This indicates that the length of* C. albicans* Sap8 propeptide may vary from 57 to 66 amino acids. The most frequent CAVIII fragments were alleles 10 (66.0%) and 8 (13.7%) corresponding to propeptides with 62 and 60 amino acids, respectively. The propeptide with 10 glutamines was the most frequent in all isolates but was higher in the bloodstream isolates (66.6%) and was lower in oral commensal (48.2%).* C. albicans* is a diploid species, and the most frequent genotypes were, as expected, 10-10 (35 strains, 32.4%) and 8–10 (30 strains, 27.8%).

**Figure 3:**
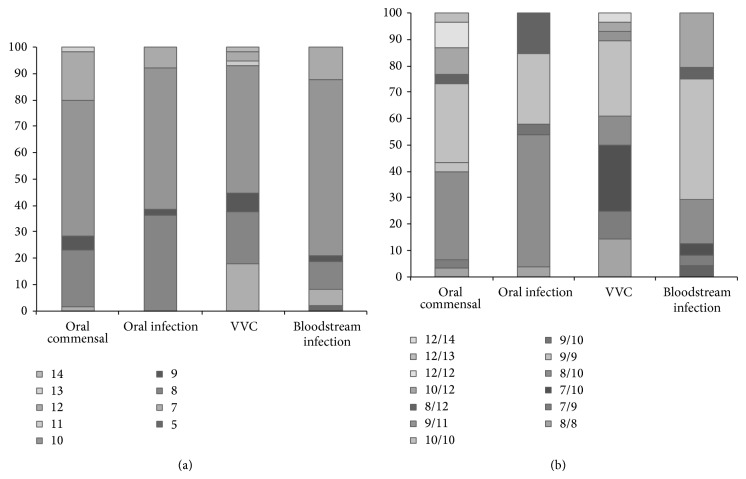
Allelic (a) and genotypic (b) frequencies of CAVIII microsatellite observed in each type of infection.

Strains were then grouped according to the type of infection and differentiation tests performed concerning allelic and genotypic distribution by testing the null hypothesis Ho: “the allelic/genotype distribution is identical across groups.” In order to select different isolates, strains were typed with CAI microsatellite marker, the most polymorphic microsatellite described for* C. albicans*, and four groups were defined, the VVC, the BI, the OI, and the OC. Differentiation tests showed significant differences (*P* < 0.05) concerning allelic and genotypic distribution in the comparison of strains from all groups except for OC versus OI, as well as BSI versus OC (Table 2). At Sap8 loci, OC strains presented the highest gene diversity (0.918) and strains from oral infections the lowest (0.676), reflecting a reduction in the gene diversity from commensalism to infection. The alleles that were not found during oral infection were 7 and 13; however, these were identified only once in commensal isolates; thus no significant differences were observed. However, allele 12 was more represented in the group of commensal isolates (identified 11 times) than in infecting strains (identified 4 times). Gene diversity of VVC strains and BSI isolates was 0.84 and 0.74, respectively. Figures 3(a) and 3(b) present the allelic and genotypic distribution in each group, showing the observed genetic diversity differences.

**Figure 4:**
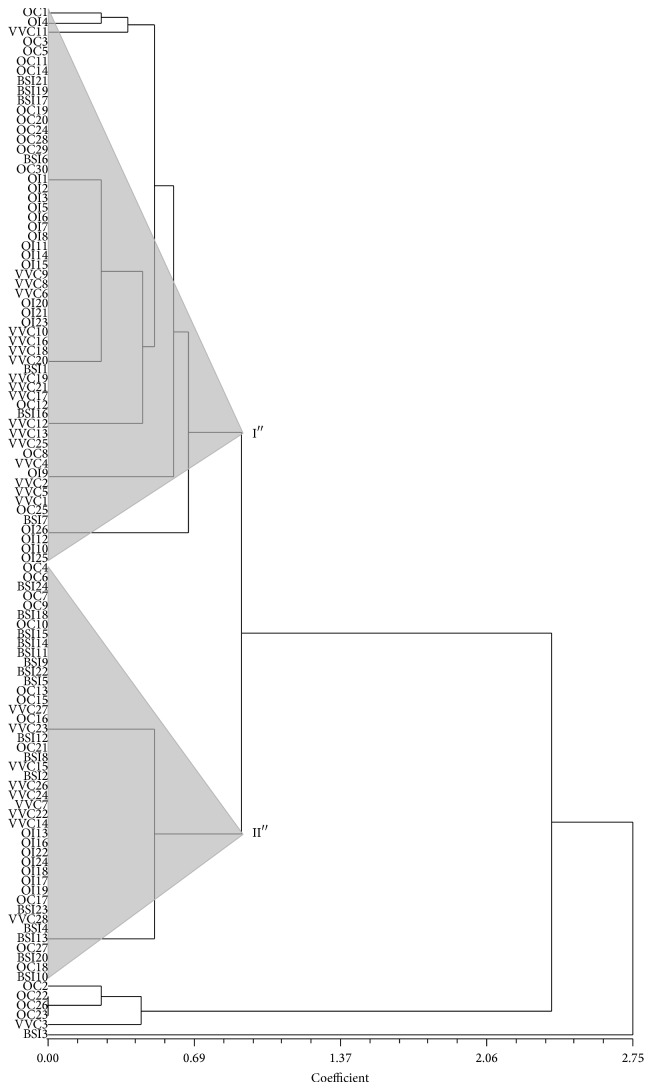
UPGMA clustering of the 174* C. albicans* isolates based on CAVIII microsatellite genotypes.

Clustering of* C. albicans* strains considering CAVIII genotypes divided them into two major groups (Figure 4). Group I included 53.7% (58 strains) of all strains while group II included 40.7% (44 strains). Strains from oral commensal were equally distributed in both groups. However, 73.1% of the strains isolated from oral infections and 64.3% from VVC were present within group I, while 66.7% of strains isolated from BSI were distributed in group II. This difference was mainly due to the fact that the majority of the strains from bloodstream infections presented genotypes, 10-10 and 10–12, clustered in group II, while isolates from oral infection presented genotypes 8–10, clustered in group I.

## 4. Discussion

Secreted aspartyl proteases are among the most studied virulence factors in* C. albicans*.* SAP* genes encode pre-pro-enzymes consisting of a signal sequence followed by a propeptide and the mature proteinase domain. Sequence analyses of the 10 members of this gene family revealed that only Sap8 presents a tract of repeated amino acids in its coding region that corresponds to a microsatellite in DNA sequence. Sap7 and Sap9 also present small tracts of repeated amino acids, but no correspondence with a mutable microsatellite was detected in their DNAs. This microsatellite is located within the propeptide region of the protein, which is essential not only for the correct folding and activity of the enzyme but also for its correct secretion. It was demonstrated that, for Kex2, the protein responsible for Saps' enzymatic activation, the accessibility and/or secondary structure of the cleavage site are essential for substrate processing [33]. Additionally, Beggah et al. [9] showed that the maturation of the recombinant* C. albicans* Sap1p expressed in* Pichia pastoris* is directed through a combination of intra- and intermolecular pathways in a dimer conformation. Thus, due to the possible implications of the polymorphism at this essential fragment it was important to assess its diversity in* C. albicans*.

Our study identified nine propeptides with different lengths for Sap8 combined into 14 genotypes. This indicates that* C. albicans* Sap8 has different propeptides with different combinations, which may render different efficacies to the proenzyme processing mechanism. A significant difference was observed between oral and vaginal isolates and considering strains from an infection process, the VVC were the ones with the highest gene diversity. A significant difference between oral, vaginal, and bloodstream environments is their pH values, in which vaginal environment has the lower pH, suitable for Sap8 activity [34]. It has been described for various aspartic proteases that the removal of the propeptide is dependent on environmental factors as well as of the prosegment structure [35]. So, the higher propeptides variability observed in strains from vaginal isolates may result from the dependence of Sap alleles for acidic environments. Indeed,* SAP8* expression has been associated with human mucosal infections but its expression was more frequent during vaginal infections than oral infections or in carriers [13, 20]. Another possible explanation would be the pH autoactivation of secreted proenzymes that were not completely processes due to a less effective propeptide; this would make any propeptide suitable for Sap8 activation in this environment. Indeed, autoproteolysis has been shown for activation of secreted* C. albicans* pro-Sap1 by reducing the pH [10, 36]. However, further studies are needed to explore these hypotheses.

Curiously, a reduction in propeptide variability was observed comparing isolates from oral commensalism with isolates from oral infections. This observation is in agreement with the finding that during infection there is a selection of strains that are able to shift to pathogenicity or resist to antifungal treatments [37]. Considering that allele 12 was the one with a significant frequency reduction in the transition from commensalism to infection we may consider that strains harboring allele 12 are not the best fitted to infection. Curiously, genotype 12-12 was observed only in oral commensal strains.

Clustering of the strains highlighted the differences in CAVIII genotype distribution particularly of strains from bloodstream infection, in which the majority of the strains presented genotype 10-10, clustering within the same group. As a consequence, the bloodstream isolates in this study presented a lower gene diversity, as observed in other studies, not only with* C. albicans* isolates [38] but also with* C. glabrata* [32].

Genes containing multiple coding mini- or microsatellite repeats are highly dynamic components of genomes and may be important as fitness determinants. In* C. albicans* a few microsatellites in coding regions have been identified and characterized such as ERK1 locus [39], genes ZNF1, CCN1, CPH1, EFG1, and MNT2 [40], but high allelic diversity has been assessed for CEK1, HYR1, HYR2, RLM1, and the ALS family [41–45]. To our knowledge, only one study reported the presence of a repetitive region, a minisatellite, within the propeptide region of a yeast protease, the Vacuolar Carboxypeptidase Y (CpY) of* Schizosaccharomyces pombe* [46]. In the former study, only one variant of CpY was observed, so the microsatellite within* C. albicans* Sap8 propeptide is the most variable described so far.

Given the recent implication of Sap8 activity in the cleavage of the signalling glycoprotein Msb2 and Cek1 MAPK pathway, we believe that the description of the genetic variability of Sap8 propeptides is important and may add a new dimension to the variability of* C. albicans* responses. This study is the first to describe the existence of different propeptides for activation of a* C. albicans* secreted aspartyl proteinase. Variability of Sap8 propeptides may be associated with the environment from which the strain was isolated.

## Supplementary Material

Description of all C. albicans strains used in this study, including the CAI and CAVII genotypes of each strain as well as the body location and the country of isolation.

## Figures and Tables

**Table 1:** Alleles structure of CAVIII microsatellite. The consensus sequence obtained from database sequence for SC5314 strain is indicated and contains 10 repetitive units.

**CAVIII—consensus sequence:** P1(25 bp)tgaaaaagttgtctcattagattttaccgttaccagaaaaccttttaatgctactgctcatggacaacatcatcaatccCAA(CAG)_3_(CAA)_6_ccagctcaaaaaagaggaactgtt caaacaagtttgattaatgaaggtccatcatatgctgctaccatcactgttggttcaaacaaacaacaacaaactgttattgttgacacaggttc-P2(25 bp)		

Allele (bp)		
**5**	(263)	Data not analysed
**6**	(266)	Data not analysed
**7**	(269)	Data not analysed
**8**	(272)	(79 bp) –––––––(CAA)_8_–––––––––(119 bp)
**9**	(275)	(79 bp) –––––––(CAA)_9_–––––––––(119 bp)
**10a**	(278)	(79 bp) CAA(CAG)_3_(CAA)_6_–––––––––(119 bp)
**10b**	(278)	(79 bp) CAA(CAG)_4_(CAA)_5_–––––––––(119 bp)
**11**	(281)	Data not analysed
**12a**	(284)	(79 bp) CAA(CAG)_3_(CAA)_8_–––––––––(119 bp)
**12b**	(284)	(79 bp) CAA(CAG)_2_(CAA)_5_CAG(CAA)_3_––(119 bp)
**13**	(287)	(79 bp) CAA(CAG)_3_(CAA)_9_–––––––––(119 bp)
**14**	(290)	Data not analysed

P1 and P2 represent the forward and reverse primers, respectively.

Data not analysed indicates not sequenced alleles.

**Table 2:** Significance of unbiased *P* values of the probability test obtained for each population pair considering microsatellite data. This test was estimated by the Fisher method (+ when *P* < 0.05 and − when *P* > 0.05).

Genotypic	Genetic			
	OI	VVC	BSI	OC
OI		0.002	0.001	0.346
VVC	0.006		0.001	0.001
BSI	0.006	0.008		0.051
OC	0.633	0.008	0.192	

VVC: vulvovaginal candidiasis; BI: bloodstream isolates; OI: oral infections; OC: oral commensal.
