# Supplementary material for: Genetic Variability of Candida albicans Sap8 Propeptide in Isolates from Different Types of Infection
Source: Biomed Res Int. 2015 Feb 4;2015:148343. doi: 10.1155/2015/148343 (PMC4334858; doi:10.1155/2015/148343)
Supplement: Supplementary file 1 — Description of all C. albicans strains used in this study, including the CAI and CAVII genotypes of each strain as well as the body location and the country of isolation. [file 148343.f1.docx]

Supplementary Table 1. CAI, CAVIII genotypes and origin of the *Candida albicans* isolates.

| **Code** | **Strain** | **Origin** | **Country** | **CAI genotype** | **CAVIII genotype** |
| --- | --- | --- | --- | --- | --- |
| OC1 | S008 | Oral cavity | Portugal | 23 30 | 09 09 |
| OC2 | S020 | Oral cavity | Portugal | 18 34 | 12 13 |
| OC3 | S040 | Oral cavity | Portugal | 26 34 | 08 10 |
| OC4 | S046 | Oral cavity | Portugal | 22 26 | 10 10 |
| OC5 | S092 | Oral cavity | Portugal | 21 28 | 08 10 |
| OC6 | S094b | Oral cavity | Portugal | 22 22 | 10 10 |
| OC7 | S110 | Oral cavity | Portugal | 24 25 | 10 10 |
| OC8 | S111 | Oral cavity | Portugal | 26 27 | 08 08 |
| OC9 | S134 | Oral cavity | Portugal | 25 26 | 10 10 |
| OC10 | S140 | Oral cavity | Portugal | 24 26 | 10 10 |
| OC11 | S142 | Oral cavity | Portugal | 27 38 | 08 10 |
| OC12 | S143 | Oral cavity | Portugal | 23 28 | 07 09 |
| OC13 | S144 | Oral cavity | Portugal | 23 25 | 10 10 |
| OC14 | S148 | Oral cavity | Portugal | 21 26 | 08 10 |
| OC15 | DM-1 | Oral cavity | UK | 26 32 | 10 10 |
| OC16 | DM-2 | Oral cavity | UK | 25 31 | 10 10 |
| OC17 | ND-1 | Oral cavity | UK | 17 39 | 10 12 |
| OC18 | ND-2 | Oral cavity | UK | 16 37 | 10 12 |
| OC19 | RB-2 | Oral cavity | UK | 28 41 | 08 10 |
| OC20 | RB-3 | Oral cavity | UK | 27 40 | 08 10 |
| OC21 | SAR1 | Oral cavity | UK | 23 27 | 10 10 |
| OC22 | S001 | Oral cavity | Portugal | 25 33 | 12 12 |
| OC23 | S005 | Oral cavity | Portugal | 25 25 | 12 12 |
| OC24 | S009 | Oral cavity | Portugal | 28 38 | 08 10 |
| OC25 | S036b | Oral cavity | Portugal | 26 32 | 08 12 |
| OC26 | S052a | Oral cavity | Portugal | 26 26 | 12 12 |
| OC27 | S076 | Oral cavity | Portugal | 23 23 | 10 12 |
| OC28 | S151 | Oral cavity | Portugal | 30 49 | 08 10 |
| OC29 | S152 | Oral cavity | Portugal | 17 17 | 08 10 |
| OC30 | S153 | Oral cavity | Portugal | 28 48 | 08 10 |
| OI1 | 2247 | Oral infection | Brazil | 25 43 | 08 10 |
| OI2 | 2250 | Oral infection | Brazil | 47 49 | 08 10 |
| OI3 | 2251 | Oral infection | Brazil | 11 20 | 08 10 |
| OI4 | 2252 | Oral infection | Brazil | 26 33 | 09 10 |
| OI5 | 2253 | Oral infection | Brazil | 27 30 | 08 10 |
| OI6 | Guy331-1 | Oral infection | UK | 48 48 | 08 10 |
| OI7 | Guy775-1 | Oral infection | UK | 42 42 | 08 10 |
| OI8 | Guy778-w1 | Oral infection | UK | 30 32 | 08 10 |
| OI9 | F | Oral infection | Portugal | 23 26 | 08 08 |
| OI10 | J | Oral infection | Portugal | 26 34 | 08 12 |
| OI11 | S | Oral infection | Portugal | 21 25 | 08 10 |
| OI12 | AA | Oral infection | Portugal | 21 22 | 08 12 |
| OI13 | Q | Oral infection | Portugal | 30 31 | 10 10 |
| OI14 | AH | Oral infection | Portugal | 27 45 | 08 10 |
| OI15 | M | Oral infection | Portugal | 28 28 | 08 10 |
| OI16 | AP | Oral infection | Portugal | 23 28 | 10 10 |
| OI17 | AN | Oral infection | Portugal | 35 37 | 10 10 |
| OI18 | CD1 | Oral infection | Portugal | 19 19 | 10 10 |
| OI19 | BH | Oral infection | Portugal | 21 24 | 10 10 |
| OI20 | N1 | Oral infection | Portugal | 27 47 | 08 10 |
| OI21 | O1 | Oral infection | Portugal | 27 46 | 08 10 |
| OI22 | V1 | Oral infection | Portugal | 30 30 | 10 10 |
| OI23 | BL | Oral infection | Portugal | 28 29 | 08 10 |
| OI24 | BP | Oral infection | Portugal | 25 35 | 10 10 |
| OI25 | BQ | Oral infection | Portugal | 34 34 | 08 12 |
| OI26 | BV | Oral infection | Portugal | 26 26 | 08 12 |
| VVC1 | 1C | Vaginal exsudate | Portugal | 17 21 | 08 08 |
| VVC2 | 2C | Vaginal exsudate | Portugal | 26 33 | 08 08 |
| VVC3 | 3C | Vaginal exsudate | Portugal | 20 37 | 12 14 |
| VVC4 | 5C | Vaginal exsudate | Portugal | 18 18 | 08 08 |
| VVC5 | 9C | Vaginal exsudate | Portugal | 24 26 | 08 08 |
| VVC6 | 10C | Vaginal exsudate | Portugal | 27 49 | 08 10 |
| VVC7 | 13C | Vaginal exsudate | Portugal | 20 27 | 10 10 |
| VVC8 | 14C | Vaginal exsudate | Portugal | 39 46 | 08 10 |
| VVC9 | 19C | Vaginal exsudate | Portugal | 27 27 | 08 10 |
| VVC10 | 27C | Vaginal exsudate | Portugal | 16 27 | 07 10 |
| VVC11 | 35C | Vaginal exsudate | Portugal | 21 25 | 09 11 |
| VVC12 | 36C | Vaginal exsudate | Portugal | 11 18 | 07 09 |
| VVC13 | 39C | Vaginal exsudate | Portugal | 20 28 | 07 09 |
| VVC14 | 45C | Vaginal exsudate | Portugal | 11 18 | 10 10 |
| VVC15 | 46C | Vaginal exsudate | Portugal | 27 27 | 10 10 |
| VVC16 | 49C | Vaginal exsudate | Portugal | 21 26 | 07 10 |
| VVC17 | 51C | Vaginal exsudate | Portugal | 22 34 | 07 10 |
| VVC18 | 53C | Vaginal exsudate | Portugal | 27 47 | 07 10 |
| VVC19 | 55C | Vaginal exsudate | Portugal | 27 42 | 07 10 |
| VVC20 | 57C | Vaginal exsudate | Portugal | 11 28 | 07 10 |
| VVC21 | 58C | Vaginal exsudate | Portugal | 28 47 | 07 10 |
| VVC22 | 3J | Vaginal exsudate | Portugal | 17 23 | 10 10 |
| VVC23 | 12J | Vaginal exsudate | Portugal | 18 25 | 10 10 |
| VVC24 | 17J | Vaginal exsudate | Portugal | 21 21 | 10 10 |
| VVC25 | 27J | Vaginal exsudate | Portugal | 22 23 | 07 09 |
| VVC26 | 29J | Vaginal exsudate | Portugal | 20 20 | 10 10 |
| VVC27 | 37J | Vaginal exsudate | Portugal | 18 27 | 10 10 |
| VVC28 | 39J | Vaginal exsudate | Portugal | 21 22 | 10 12 |
| BSI1 | HSJ 63 | Blood | Portugal | 16 25 | 07 10 |
| BSI2 | HSJ 69 | Blood | Portugal | 16 25 | 10 10 |
| BSI3 | HSJ 93 | Blood | Portugal | 21 25 | 05 07 |
| BSI4 | HSJ 114 | Blood | Portugal | 12 12 | 10 12 |
| BSI5 | HSJ 124a | Blood | Portugal | 18 34 | 10 10 |
| BSI6 | HSJ 140 | Blood | Portugal | 18 34 | 08 10 |
| BSI7 | HSJ 144 | Blood | Portugal | 18 34 | 08 12 |
| BSI8 | HSJ 130 | Blood | Portugal | 12 12 | 10 10 |
| BSI9 | HSJ 141 | Blood | Portugal | 12 17 | 10 10 |
| BSI10 | HSJ 154 | Blood | Portugal | 26 26 | 10 12 |
| BSI11 | HSJ 155 | Blood | Portugal | 29 29 | 10 10 |
| BSI12 | HSJ 164 | Blood | Portugal | 26 28 | 10 10 |
| BSI13 | HSJ 165 | Blood | Portugal | 21 25 | 10 12 |
| BSI14 | IPOL 2 | Blood | Portugal | 25 25 | 10 10 |
| BSI15 | IPOL 7 | Blood | Portugal | 26 26 | 10 10 |
| BSI16 | IPOL 11 | Blood | Portugal | 23 27 | 07 09 |
| BSI17 | IPOL 13 | Blood | Portugal | 40 40 | 08 10 |
| BSI18 | IPOL 14 | Blood | Portugal | 20 28 | 10 10 |
| BSI19 | IPOL 15 | Blood | Portugal | 35 44 | 08 10 |
| BSI20 | IPOL 17 | Blood | Portugal | 16 38 | 10 12 |
| BSI21 | IPOL 19 | Blood | Portugal | 18 28 | 08 10 |
| BSI22 | IPOL 20 | Blood | Portugal | 21 25 | 10 10 |
| BSI23 | IPOL 21 | Blood | Portugal | 19 34 | 10 12 |
| BSI24 | IPOL 22 | Blood | Portugal | 21 26 | 10 10 |

**VE** Vaginal Exudate; **OI** Oral Infection; **UK** United Kingdom
